# Explicit Motor Imagery for Grasping Actions in Children With Spastic Unilateral Cerebral Palsy

**DOI:** 10.3389/fneur.2019.00837

**Published:** 2019-08-07

**Authors:** Antonino Errante, Francesca Bozzetti, Silvia Sghedoni, Barbara Bressi, Stefania Costi, Girolamo Crisi, Adriano Ferrari, Leonardo Fogassi

**Affiliations:** ^1^Department of Medicine and Surgery, University of Parma, Parma, Italy; ^2^Neuroradiology Unit, Department of Diagnostic, University Hospital of Parma, Parma, Italy; ^3^Azienda Unità Sanitaria Locale – IRCCS of Reggio Emilia, Reggio Emilia, Italy; ^4^Department of Neuroscience, University of Modena and Reggio Emilia, Modena, Italy

**Keywords:** cerebral palsy, motor imagery, mental chronometry, fMRI, motor simulation, grasping

## Abstract

**Background:** Motor Imagery (MI) refers to mental simulation of a motor action without producing any overt movement. Previous studies showed that children with Unilateral Cerebral Palsy (UCP) are impaired in implicit MI, as demonstrated by the performance of Hand Laterality Judgment tasks. The aim of this study was to examine the specificity of explicit MI deficits in UCP children.

**Methods:** A group of UCP children (*n* = 10; aged 9–14) performed a mental chronometry task consisting in grasping an object and placing it into a container, or in imagining to perform the same action. As control, a group of typically developing (TD) children, matched by age, performed the same task. Movement durations for executed and imagined trials were recorded. A subgroup of 7 UCP children and 10 TD children also underwent a session of functional MRI to examine the activation of parieto-frontal areas typically associated to MI processes, during the imagination of reaching-grasping actions performed with the paretic hand.

**Results:** Behavioral results revealed the existence of a correlation between executed and imagined movement durations both in TD and UCP groups. Moreover, the regression analysis in TD children showed that higher scores in mental chronometry tasks were positively correlated to increased bilateral activation of the intraparietal sulcus (IPS), superior parietal lobule (SPL), and dorsal premotor (PMd) cortex. A similar analysis revealed in the UCP group a positive correlation between a higher score in the mental chronometry task and bilateral activations of IPS, and to activation of contralesional, right PMd, and putamen during imagination of grasping movements.

**Conclusions:** These results provide new insights on the relationship between MI capacity and motor deficits in UCP children, suggesting the possibility of the use of explicit MI training to improve patient's upper limb motor functions.

## Introduction

Cerebral palsy (CP) is a group of permanent disorders of the development of movement and posture, causing activity limitations, that are attributed to non-progressive disturbances occurred in the developing fetal or infant brain (1). CP is the most common physical disability in childhood and occurs in 1 out of 500 live births (2). Children with unilateral cerebral palsy (UCP), in which only one side of the body is involved, represent 38% of CP (3). UCP can be attributed to perinatal ischemic stroke or, in premature infants, to malformations of white-matter, producing unilateral porencephalic cavities (or cysts) (4, 5). In UCP, the upper limb is generally more affected than the lower one (6). About the two upper limbs, some evidence showed, by evaluating motor dexterity and precision grip or complex reaching grasping actions, that also the hand considered not affected has actually some deficits (7, 8). However, unimanual activities are generally performed by the less affected upper hand, while daily life ones, which are prevalently bimanual, can be severely impaired (9, 10).

Several models of intervention are currently available to improve upper limb function in UCP children, such as intramuscular injections of botulinum toxin-A (BoNT-A), constraint-induced movement therapy (CIMT), and intensive hand-arm bimanual training (BIM) (11). However, in the last decade, it has been shown that motor deficits in UCP children are not only related to problems in overt movement execution, but can also be associated to deficits in motor planning and motor imagery (MI) (12). This latter consists in the internal simulation of a movement/action without its overt execution (13, 14). Neuroimaging evidence in humans has repeatedly demonstrated (15–18) that sensorimotor circuits involved in performing real movements/actions, including supplementary motor area (SMA), dorsal and ventral premotor cortices (PMd, PMv), inferior parietal lobule (IPL), superior parietal lobule (SPL), prefrontal areas, cingulate cortex, and cerebellar regions, are activated also during performance of MI of the same movements/actions.

In recent years, the application of MI as a tool for recovery of motor function in adult patients (19–21) and children with motor developmental coordination disorder (DCD) (22, 23) has suggested the possibility of using a similar rehabilitation proposal in UCP children (24). However, accumulated evidence has revealed that often action planning and MI can be severely compromised in UCP patients (25, 26). Behaviorally, a common method for testing if a patient is able to evoke MI is the Parsons' Hand Laterality Judgment (HLJ) task (27): pictures of hands are presented in different orientations and participants have to provide a laterality judgment. The task is designed to test a specific type of MI, namely implicit MI, based on the fact that participants have to imagine rotating their own hand to match it with the observed one. There is considerable consensus that in the period ranging from 5 and 12 years, TD children increase their accuracy and speed in solving this type of task (28–30).

However, recent studies (31, 32) have proposed that implicit tasks, as the HLJ, are not the most appropriate in assessing MI ability in UCP patients. Indeed, mental rotation by itself is not sufficient to conclude if patients are able to evoke an effective MI strategy. They may use alternative strategies, for instance a visual strategy, that is the rotation of the hand imagined from a third-person perspective, or apply an abstract rule to make the laterality judgment. Moreover, patients with a body-awareness deficit, as UCP ones, may be unable to produce a kinesthetic image of motor action, but may be facilitated by using an explicit paradigm, such as mental chronometry (13, 33–35). Usually, in this latter paradigm, participants are instructed to actually perform a simple movement (pointing task) and, in a separate block, to imagine performing the same movement. A high correspondence between actual and imagined movement duration is taken as evidence of MI ability (34, 36–38). Overall, the studies employing the mental chronometry paradigm indicate that children's ability to engage MI gradually increases until at least 12 years of age, as demonstrated by age-related increases in temporal congruence and compliance with Fitts' law for the imagined task (33, 35, 39). However, the finding of temporal congruence by itself does not allow to establish if effective MI occurred, because participants may also have used alternative non-motor imagery strategies or even counting to solve the task. To address this issue, an often-used method is to manipulate task difficulty and examine the effects on both actual and imagined performance. For instance, studies employing pointing tasks (28, 33, 35, 40) found a compliance with Fitts' law (41), according to which movement duration is logarithmically related to task difficulty. Using this type of control, several studies on TD children found that the linear fit in the MI task increased with age: the 6- to 7-years-old children showed a lower fit than 10- to 16- years-old ones (33, 35).

To date, a mental chronometry paradigm has never been used to study the relationship between execution and imagination of grasping actions in UCP children. The aim of the present study was to investigate the processes underlying explicit MI ability for grasping actions in UCP children, using a combined behavioral and neuroimaging approach. A homogeneous group of UCP children with selected features of upper limb motor deficits, and a group of age-matched TD participants performed a mental chronometry task, requiring them to execute or imagine to perform reaching-grasping actions. We hypothesized that, if participants were able to use a motor strategy (compared to visual strategies, counting etc.), a significant correlation between durations of the execution and imagination tasks should be observed. A sub-group of UCP children and a subgroup of TD children also underwent a session of functional MRI to examine brain activation during the performance of an explicit MI task, consisting in imagining a reaching-grasping action similar to the one they performed during the mental chronometry task. The aims of this neuroimaging investigation were: (a) to describe the activation of sensorimotor parieto-frontal areas of UCP and TD children during the performance of a MI task; (b) to correlate the behavioral data provided by the mental chronometry task to specific activation patterns in parietal and premotor regions. We hypothesized that those children with UCP showing higher scores in the behavioral task should have increased activation of the above-mentioned parieto-frontal areas during the performance of the MI fMRI task, with respect to those with lower scores.

## Materials and Methods

### Participants

Ten children with confirmed diagnosis of unilateral brain lesion (5 males; range 9–14 years old; M = 12.3; SD = 1.88) and clinical congenital hemiplegia (Group: UCP children; see Table 1) were selected starting from a large sample (*N* > 150) of hemiplegic children in the IRCCS S. Maria Nuova Hospital (Reggio Emilia, Italy) database, according to the following inclusion criteria: (1) confirmed diagnosis of UCP according to definition (MRI and clinical history); (2) age 9–14 at time of recruitment; (3) mild or moderate upper-limb disability, i.e., active use of affected upper limb ranging from poor active assisted use to complete spontaneous use, according to House Functional Classification (HFC) system (42, 43) with grades between 4 and 5; (4) no cognitive, visual or auditory impairments; (5) no history of seizures or seizures well-controlled by therapy.

**Table 1:** Demographic data, clinical features and functional classification in cases group.

**UCP**	**Sex**	**Age**	**Lesion side**	**GA**	**CP Type**	**Pathophysiology**	**HFC**	**MACS**	**Manipulation Pattern**	**fMRI**
#1	F	13	LH	32	Right UCP	Periventricular-intraventricular hemorrhage (PIVH)	5	3	Synergic Hand	Yes
#2	M	14	LH	38	Right UCP	Hypoxic-ischemic pre-natal brain injury	5	3	Synergic Hand	Yes
#3	M	9	LH	40	Right UCP	Factor V deficiency	5	3	Synergic Hand	Yes
#4	F	13	LH	36	Right UCP	Posterior cerebral artery ischaemic stroke (distal branches)	4	3	Synergic Hand	Yes
#5	M	10	LH	37	Right UCP	Middle cerebral artery ischaemic stroke (distal branches)	5	3	Synergic Hand	Yes
#6	M	10	LH	30	Right UCP	Hypoxic-ischemic pre-natal brain injury in pre-term infant	5	3	Synergic Hand	Yes
#7	M	14	LH	36	Right UCP	Middle cerebral artery ischaemic stroke (deep and distal branches) and water-shed territories	5	3	Synergic Hand	Yes
#8	F	13	RH	39	Left UCP	Perinatal ischemic stroke	5	2	Semi-functional Hand	NA
#9	F	14	RH	32	Left UCP	Periventricular-intraventricular (subependymal) hemorrhage (PIVH)	4	3	Synergic Hand	NA
#10	F	13	RH	36	Left UCP	Perinatal ischemic stroke	5	2	Semi-functional Hand	NA

*F, Female; GA, Gestational Age; HFC, House Functional Classification level; LH, Left Hemisphere; M, Male; MACS, Manual Ability Classification System; NA, Not Available; RH, Right Hemisphere; UCP, Unilateral Cerebral Palsy*.

As control, a group of right handed (44) TD children matched by age (*n* = 12; 6 males; range 9–14 years old) were recruited.

A sub-group of 7 UCP children and a sub-group of 10 TD children, matched by age, were also involved in a functional MRI study aimed at investigating the neural correlates of explicit MI for grasping actions and at comparing brain activations with the results provided by the behavioral experiment using supplementary inclusion/exclusion criteria, namely: (1) sufficient cooperation to perform imaging studies for 15 min; (2) no exclusions for 3-Tesla Magnetic Resonance System such as metal implants, shunts, etc. The fact that the three participants who did not undergo the neuroimaging investigation were all with left hemiparesis was just due to these latter criteria, and not purposefully planned.

The protocol was approved by the Institutional Review Board of the University of Parma and the Ethics Committee of University of Parma (Study ID: UNIPRMR750c1). Parental written informed consent for all participants in accordance with the Declaration of Helsinki was obtained.

### Clinical Assessment of UCP Children

All UCP children participating in this study were able to understand the given instructions and did not present major cognitive deficit, as indicated by direct observation, IQ (if available) >/ = 70 assessed using the Wechsler Preschool and Primary Scale of Intelligence–Fourth Edition [WPPSI; (45)] or attendance of a mainstream primary school.

Motor deficits were classified according to the House Functional Classification (HFC) system (42, 43). It was originally developed for the evaluation of the affected hand function after surgery for thumb-in-palm deformity in children with spastic UCP and has been used to evaluate children before and after upper extremity botulinum toxin-A injections (46, 47). The classification consists of nine levels, ranging from a hand that is not used at all (grade 0) to one that is used spontaneously and independently from the other hand (grade 8). Furthermore, the Manual Ability Classification System (MACS) was also used to categorize UCP children into five levels, according to their ability to perform manual activities in everyday life (48). Note that in the MACS low scores correspond to higher ability.

A further classification of UCP children is based on the description of five patterns of manipulation by analyzing hand kinematic profile and functional use (49). On the basis of this classification, children participating in this study use their non-preferred upper limb by means of *semi-functional* or *synergic* strategies:

The semi-functional hand is characterized by the presence of a termino-lateral pinch with substantially adduced thumb. Orientation, anticipation and pre-adaptation of the hand are possible, but uncertain. The object can be promptly passed from one hand to the other, but its release is rough, with frequent need of visual control. This pattern shows, during bimanual activities, a good cooperation between the two hands. The non-preferred hand is used spontaneously as first only when the patient acts in the extreme part of the hemispace ipsilateral to this hand.

The synergic hand is characterized by a stereotyped grasping showing flexion and extension synergies and servomotor movements in the releasing action. The object is passed with difficulties from the non-preferred hand to the preferred one, with necessity of visual control. Manipulation is extremely limited. In this pattern, there can be a collaboration in bimanual activities for achieving the same aim, with the non-preferred hand supporting the preferred one.

Individual scores and descriptions of manipulation patterns for the used classification systems are reported in Table 1.

### Mental Chronometry Task for Reaching-Grasping Actions

**Figure 1:**
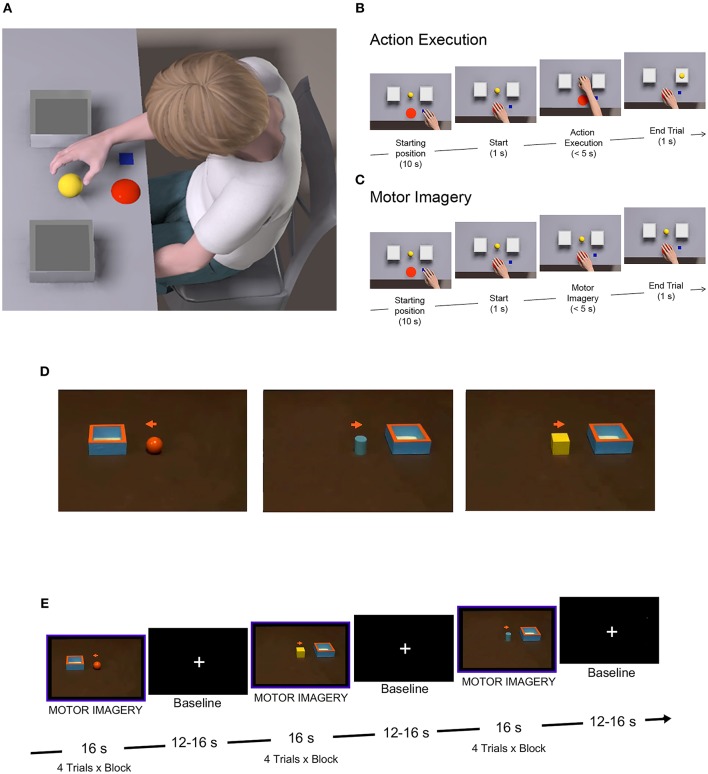
Behavioral and fMRI paradigms. **(A)** Experimental setting for the mental chronometry task. **(B)** *Action execution* trial, consisting in reaching/grasping the object, placed at one of the three different distances with the preferred hand or the non-preferred one in different trials, and placing it into a container. **(C)** *Motor imagery* trial, requiring participants to imagine performing the same action as in **(B)**, from a first-person perspective. **(D)** Stimuli used during the fMRI task, showing an object and a box on a table. Simultaneously, participants had to imagine themselves grasping the object with the non-preferred hand and placing it into the box. **(E)** fMRI block design, alternating task and rest conditions, with a total number of eight blocks, constituted by four trials per block.

All participants performed a mental chronometry task for reaching-grasping actions. The task was performed using a wooden support (60 × 40 cm) with two boxes (6 × 6 × 4 cm) positioned at different distances (D1 = 100 mm, D2 = 150 mm, D3 = 200 mm) from a central press button (Figure 1A). Between the two boxes, a target object was positioned, consisting of a little plastic sphere (diameter 35 mm). Subjects were seated in front of a table with their hand resting on the starting position. Participants were explicitly asked to perform two tasks: (a) *action execution*, consisting in reaching/grasping the object, placed at one of three different distances with their preferred or non-preferred hand in different trials, and placing it into a container (Figure 1B); (b) *motor imagery*, requiring participants to imagine performing the same action as in (a) from a first-person perspective (Figure 1C). The preferred hand corresponded to the dominant hand of TD participants and to the less-affected hand of UCP children. For the action execution task, at the beginning of each trial, participants, verbally instructed by the experimenter, waited for the go-signal (BEEP sound; duration 500 ms), then they had to press the central button, perform the action and then return to the button and press it again to stop the trial. Participants were required to perform the action as fast and accurately as possible. For the motor imagery task, participants were required to press the central button and imagine performing the action, without doing it, while the hand remained immobile near the button. Then, they had to press the button again to stop the trial. Subjects were supposed to feel as if they were actually moving their hand in a first-person perspective for reaching-grasping the object. Specifically, they always kept their eyes open during task performance, to avoid the formation of a visual image in a third-person perspective. In order to verify the absence of actual hand movement during the motor imagery task, all trials were video recorded and given scores by two independent observers. In both tasks, three different distances were used (100, 150, and 200 mm) in order to test the possible differences of task duration and to verify, in the MI task, that children used an effective motor strategy. Participants performed 10 trials for each distance and for each hand, for a total of 30 trials per task, administered in blocks, each constituted by 10 trials of the same task, counterbalanced across blocks. Thus, the experiment consisted of a factorial design: 2 (Task: action execution, motor imagery) × 3 (Distance: 100, 150, 200 mm) × 2 (Hand: preferred hand, non-preferred hand) × 2 (Group: UCP children, TD children) with the first three factors as repeated measures within-subject, and the last one as between-group factor.

#### Behavioral Data Analysis

For each participant, the mean duration was calculated separately for each Task, Distance and Hand used. A repeated-measures analysis of variance (rmANOVA) was conducted on mean movement durations, with Group as a between-subjects factor and Task, Distance and used Hand as repeated-measures. Pairwise comparisons were performed using *post-hoc* Bonferroni test to highlight significant findings. When assumption of sphericity was violated, Greenhouse-Geisser correction was applied.

The temporal congruence between *action execution* and *motor imagery* performance was determined by calculating individual Pearson correlation between the average overt and covert movement durations (defined as Motor Imagery Ability score—MIA). Individual correlations were then subjected to rmANOVA to test Group effects on temporal congruence.

### fMRI Study

The two sub-groups of UCP patients and TD children underwent a session of fMRI in which they were instructed to imagine reaching-grasping actions similar to those they performed during the behavioral task. Participants were presented with videoclips showing a central object (sphere, cube or cylinder) and a box (6 × 6 cm), placed on right or left with respect to the object (Figure 1D). Instructions were to observe the presented context, then a cue (a little arrow) would appear in the central part of the screen, instructing children to imagine themselves performing the action with the right non-preferred hand (to imagine grasping the object and placing it into the box located in the position cued by the arrow). A total of 32 experimental video stimuli were presented in blocks lasting 16 s each. Eight task blocks were presented in a single functional run, with 12–16 s of baseline (fixation of a central white cross) after each block (see Figure 1E). Imaging sessions lasted ~10 min.

#### MRI Scanning Procedure

Both UCP and TD children performed a training phase before the fMRI session aimed at familiarizing them with the experimental procedure. Visual stimuli were presented by means of a digital video system (60 Hz refresh rate) with a resolution of 800 horizontal pixels x 600 vertical pixels with horizontal eye field of 30° (Resonance Technology, Northridge, CA). Sound-attenuating headphones were used to muffle scanner noise and give instructions to participants. Digital transmission of signal to scanner was via optic fiber. Software E-Prime 2 Professional was used for stimulus presentation. Before the beginning of MRI acquisition, children received precise instructions not to make any voluntary movement during the MI task. An MR-compatible camera (acquisition frequency 60 Hz; MRC Systems) was used to video record actual hand movement or mirror movements during all experimental sessions. The absence of movements during motor imagery performance was investigated by two independent observers at the end of the scanning session.

#### MRI Data Acquisition

Anatomical T_1_-weighted, anatomical T_2_-weighted FLAIR FS-ARC, and functional T_2_*-weighted MR images were acquired with a 3-T General Electric scanner (MR750 Discovery) equipped with an 8-channel receiver head-coil. A three-dimensional (3D) high-resolution T_1_-weighted IR-prepared fast SPGR (Bravo) image covering the entire brain was acquired and used for anatomical reference. Its acquisition parameters were as follows: 196 slices, 280 × 280 matrix with a spatial resolution of 1 × 1 × 1 mm, TR = 9,700 ms, TE = 4 ms, FOV = 252 × 252 mm; flip angle = 9°. Functional volumes were acquired while participants performed the motor imagery task with the following parameters: 37 axial slices of functional images covering the whole brain acquired using a gradient-echo echo-planar imaging (EPI) pulse sequence, slice thickness = 3 mm plus interslice gap = 0.5 mm, 64 × 64 × 37 matrix with a spatial resolution of 3.5 × 3.5 × 3.5 mm, TR = 2,000 ms, TE = 30 ms, FOV = 205 × 205 mm^2^, flip angle = 90°, in plane resolution = 3.2 x 3.2 mm^2^.

#### Data Preprocessing and Statistical Analysis

Data analysis was performed with SPM12 (Wellcome Department of Imaging Neuroscience, University College, London, UK; http://www.fil.ion.ucl.ac.uk/spm) running on MATLAB 2018a (The Mathworks, Inc.). Structural images were manually centered and reoriented with functional images to the anterior-posterior commissure axis. The first four EPI volumes were discarded to allow for T_1_ equilibration effects. For each subject, all volumes were slice timing corrected, spatially realigned to the first volume and un-warped to correct for between-scan motion. Motion parameters were used as regressors of no-interest in the model to account for translation and rotation along the 3 possible dimensions as determined during the realignment procedure. Individual datasets were excluded if excessive head motion was observed (translation > 3 mm or rotation > 3°). T_1_-weighted images were segmented into gray, white matter, and cerebrospinal fluid and spatially normalized to a standard Montreal Neurological Institute (MNI) template for pediatric data from the 4.5 to 18.5 year age (50). The spatial transformation derived from this segmentation was then applied to realigned EPIs for normalization and re-sampled in 2 × 2 × 2 mm^3^ voxels using trilinear interpolation in space. All functional volumes were then spatially smoothed with an 8 mm full-width half-maximum isotropic Gaussian kernel.

The pre-processed functional data for each participant were entered in single-subject whole-brain analysis (51). Blood oxygen level dependent (BOLD) signal was modeled in a General Linear Model (GLM) by a design matrix comprising onset and duration of each event, according to experimental task. This analysis employed event-related convolution models using the hemodynamic response function (HRF) provided by the software SPM12. We used one predictor of interest that was a boxcar function with duration of motor imagery blocks, containing four trials each. Single subject activation maps were produced using a fixed-effect analysis (FFX) at a statistical threshold of *p* < 0.001 [with cluster level family-wise error (FWE) rate correction for multiple comparisons]. Anatomical description was performed on the basis of probabilistic cytoarchitectonic maps as implemented in Anatomy toolbox for SPM12 (52).

Two multiple regression analyses were performed at multi-subject level, one for UCP and one for TD group, separately, to look for a linear relationship between brain activity (BOLD signal change) during explicit MI task and MIA score (Pearson correlation coefficient) obtained in the mental chronometry paradigm task with the non-preferred hand. These regression analyses were designed to study responses across the entire brain at a threshold of *p* < 0.001, after application of FWE correction for multiple comparisons at cluster level.

#### Lesion Analysis

Lesions were manually delineated on the T_2_-weighted FLAIR images, using the MRIcron software (http://www.cabi.gatech.edu/mricro/mricro). Lesions were mapped by two expert neuroradiologists (FB and GC) delineating the boundary of the lesion directly on the image for every single transverse slice, using MRIcron. Both MRI scan and lesion shape were then mapped into stereotaxic space using the normalization algorithm provided by SPM12. After normalization, all lesions were carefully reviewed to ensure that lesion maps accurately reflected the extent of lesions in MNI space. Manual adjustments were made if necessary to better match the MNI template.

## Results

### Behavioral Results

**Figure 2:**
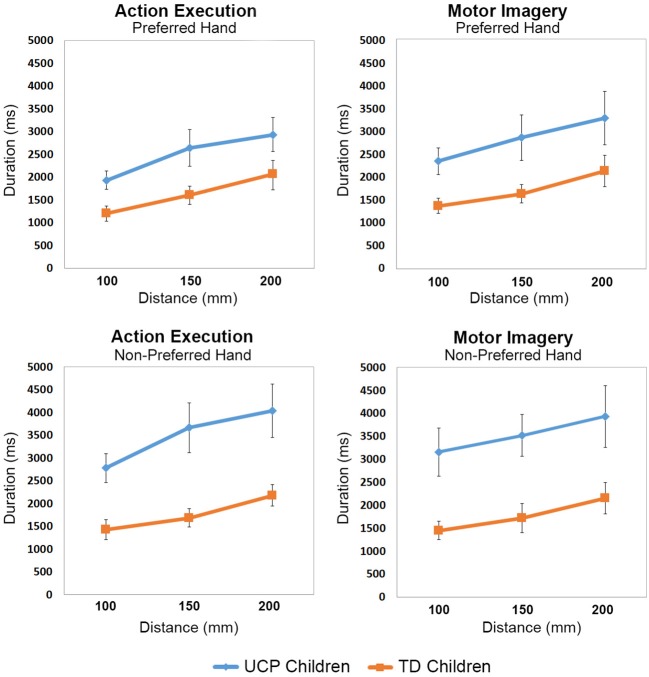
Line graphs showing mean movement durations (ms) with increased Distances for each group of participants.

Group mean duration for each distance and for preferred/non-preferred hands corresponding to the action execution and motor imagery tasks, are presented in Figure 2 (individual durations in both execution and MI tasks for the three distances in UCP children are presented in Supplementary Table 1). The rmANOVA revealed significant main effect of Group [*F*_(1, 20)_ = 38.049, *p* < 0.0001, partial η^2^ = 0.65, Task *F*_(1, 20)_ = 47.871, *p* < 0.0001, partial η^2^ = 0.70], Hand [*F*_(1, 20)_ = 45.395, *p* < 0.0001, partial η^2^ = 0.69], and Distance [*F*_(2, 40)_ = 16.524, *p* < 0.0001, partial η^2^ = 0.45]. *Post-hoc* comparison showed that UCP children took more time (*M* = 3,056 ms) to perform the action execution and motor imagery tasks compared to TD children (*M* = 1,750 ms, *p* < 0.001). In addition, mean durations for both tasks in both groups increased according to Distance, with faster responses for D1 (*M* = 2,110 ms) compared to D2 (*M* = 2,393 ms, *p* < 0.001) and for D2 compared to D3 (*M* = 2,529 ms, *p* < 0.001). Moreover, both groups of children performed the execution task faster with the preferred hand (*M* = 1,824 ms) with respect to the non-preferred hand (*M* = 2,307 ms, *p* < 0.001), while there was no difference between preferred and non-preferred hand during the MI task (PH *M* = 2,489; NPH *M* = 2,754).

The interaction Group × Hand was also significant *F*_(1, 4220)_ = 27.7841, *p* < 0.00004, partial η^2^ = 0.57. *Post-hoc* tests revealed that UCP children took less time to perform both tasks with the preferred hand (*M* = 2,698 ms) compared to non-preferred one (*M* = 3,414 ms, *p* < 0.0001), while no such difference was present in the TD children, who had similar performance with both hands.

In addition, the performance of UCP children was slower compared to that of TD children, not only when using their non-preferred hand (UCP *M* = 3,414 ms, TD *M* = 1,795 ms, *p* < 0.001), but also with the preferred one (UCP *M* = 2,698, TD M = 1,705 ms, *p* < 0.001). No other interaction between Group and other factors was significant.

**Figure 3:**
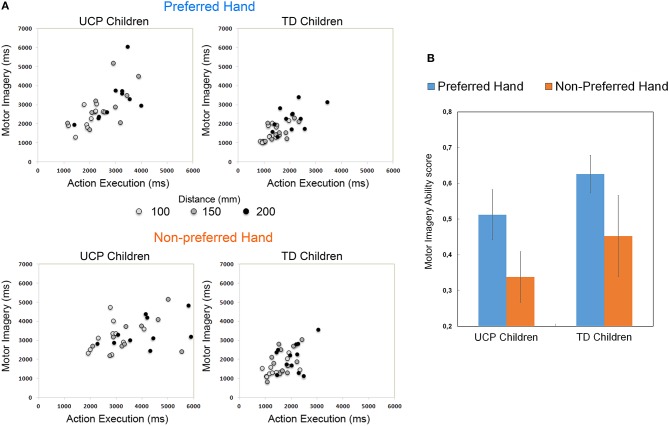
**(A)** Scatterplot showing correlations between movement durations (ms) in the Action Execution and Motor Imagery conditions performed by each group of participants, using the preferred and the non-preferred hand. The lighter dots represent the trials with the shortest distance, the darker dots those with the longest distance. **(B)** Histogram shows the Motor Imagery Ability (MIA) scores, calculated as correlation (Pearson coefficient) index between movement times under Action Execution and Motor Imagery conditions.

Scatter plots of correlations between action execution and motor imagery durations in each Group are shown in Figure 3A, for both preferred and non-preferred hand. This measure is referred to the temporal congruence between tasks and is defined as MIA score. Mean MIA score (Figure 3B) corresponding to the use of preferred hand was *r* = 0.51 for UCP children and *r* = 0.61 for TD children. Moreover, mean MIA score using the non-preferred hand was *r* = 0.33 for UCP children, and *r* = 0.45 for TD children. ANOVA results showed that the magnitude of MIA score for preferred and non-preferred hand did not differ between groups *F*_(2, 19)_ = 2.827, *p* = 0.084 (see Supplementary Table 2 for individual MIA scores of both TD and UCP participants).

### Lesion Anatomy

In all patients, the lesion was strictly unilateral (see Supplementary Figure 1). Most lesioned regions involved the periventricular and deep white matter, with the typical periventricular leukomalacia (PVL) and subcortical leukomalacia as the results of insults occurred during pre and peri-natal period. The lesions were characterized by white matter loss, gliosis, and cavitated/cystic lesions adjacent to external angles of lateral ventricles or diffuse white matter injury and hypomyelination. Both periventricular and subcortical leukomalacia are on a continuous disease spectrum: vascular border zones shift toward periphery as the brain further matures; for this reason, white matter lesions move from periventricular to subcortical zone. Considering the different archetypes of childhood hemiplegia, we can reasonably suppose that the lesions of UCP children enrolled in this study may belong to the II (Prenatal; lesion of the 3rd trimester) and III (Connatal; perinatal lesion at term) group according to the classification of childhood hemiplegia of Cioni et al. (4). The highest lesion overlap was found in subcortical white matter of left hemisphere. Conversely, cortical involvement of regions outside the periventricular zone, i.e., the inferior frontal, dorsolateral frontal, inferior and superior parietal regions was less frequently found (*n* = 2 subjects). Overall, lesion distribution was similar to previous lesion studies in CP (53, 54). Table 2 summarizes the anatomical characteristics of the lesions of all 7 UCP children. At the macroscopical examination no patient had bilateral lesions. The degree of white matter loss, and consequently of ventricular dilatation, was classified as mild, moderate, and severe based on the involvement of the periventricular, deep and subcortical white matter (WM). The degree of corpus callosum atrophy was classified as mild (<1/3), moderate (<1/3>), severe (>1/3), and ventricle (VL) dilatation as minimal, mild, moderate or severe. Only two patients showed signs of Wallerian degeneration of the cortico-spinal tract documented by smooth hyperintensities in FLAIR images. Limited marginal gliosis was present in five patients.

**Table 2:** Neuroradiological findings and anatomical characteristics of lesions in cases group.

**UCP**	**GMD**	**WMD**	**PV- WM**	**D-WM**	**SC- WM**	**VD**	**Localization**	**Gliosis**	**CC atrophy**	**WD of CS**	**TYPE**
#1	0	**x**	**x**	**x**	0	Moderate	F-P	Mild	**xx**	**Yes**	II
#2	0	**x**	**x**	**x**	**x**	Severe	F-P	No	**xxx**	No	II
#3	**x**	**x**	**x**	**x**	**x**	Mild	F-P	Mild	**x**	No	II
#4	**xx**	**x**	**x**	**x**	**x**	Moderate	I-F-P	Mild	**x**	**Yes**	III
#5	**xx**	**x**	**x**	**x**	**x**	Mild	P	Mild	**x**	No	III
#6	0	**x**	**x**	0	0	Minimal	F	No	Normal	No	II
#7	0	**x**	**x**	**x**	**x**	Moderate	F-P	Mild	**xx**	No	II

*Lesion types were defined according to the Cioni's classification (1999). CC, Corpus Callosum; CC Atrophy: X, Mild (<1/3) damage; XX, Moderate (<1/3>) damage; XXX, (>1/3) Severe damage; GMD, Gray Matter Damage (0, absent; x, mild; xx, moderate); WMD, White Matter Damage (PV-WM, Periventricular White Matter; D-WM, Deep White Matter; SC-WM, Subcortical White Matter); localization (F, frontal lobe; I, insular cortex; P, parietal lobe); VD, Ventricle Dilatation; WD, Wallerian degeneration of corticospinal tract. Bold values indicate the presence of pathological signs for all the analyzed lesions*.

### Brain Activation Results

**Figure 4:**
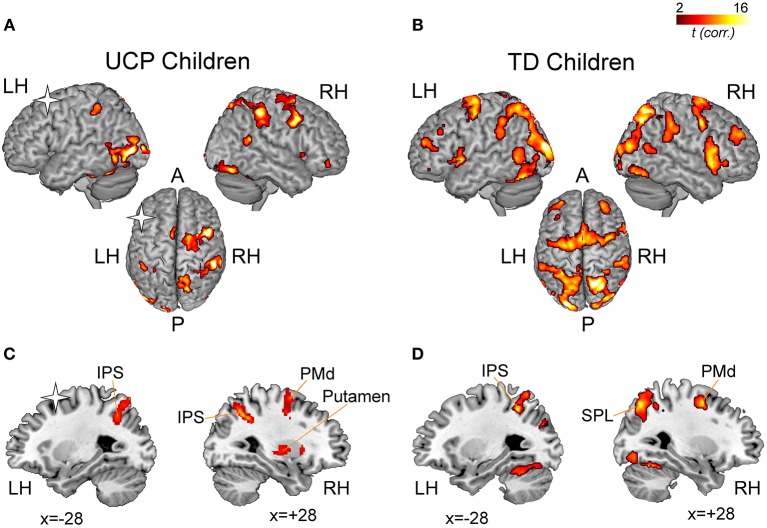
fMRI results. Statistical maps illustrating significant activations during the imagination of grasping actions in children with UCP **(A)** and in TD children **(B)**. The statistical maps are overlaid into a standard MNI template (ch2Better, MRICron). The results of the regression analysis conducted on UCP group **(C)** and TD group **(D)** indicate correlations between percent signal change in each voxel and the individual Motor Imagery Ability (MIA) score obtained during the fMRI task using the non-preferred hand. A, Anterior; LH, Left Hemisphere; P, Posterior; RH, Right Hemisphere. Asterisks indicate the lesioned hemisphere in UCP patients.

Figure 4 illustrates the fMRI results. Similar to previous studies in adults (15), the imagination of reaching, grasping and placing an object was associated to activations, in TD children, of the superior parietal lobule (SPL), the intraparietal sulcus (IPS) and the precuneus, bilaterally, plus the rostral left inferior parietal lobule (IPL). In the frontal lobes, TD children's activations included bilateral inferior frontal gyrus (IFG pars opercularis), PMd, middle frontal gyrus (MfG), dorsolateral prefrontal cortex (DLPFC), superior frontal gyrus/sulcus, and supplementary motor area (SMA). In the temporal lobe, activations included posterior superior and middle temporal gyri (pSTG/MTG). Consistently activated subcortical regions were left putamen, pallidum, and right thalamus and the lobule VI of the cerebellum, bilaterally (Figure 4B).

In UCP children, imagining to reach, grasp and place an object activated, in the contralesional hemisphere, the IPL, IPS, SPL, and STG/MTG. In the contralesional frontal lobe, other regions consistently activated were dorsal and ventral premotor cortices (PMd, PMv), MfG, SMA, and DLPFC. More than four patients showed consistent activation within the basal ganglia (putamen and pallidum). In the ipsilesional, left hemisphere, patients #1, #2, and #6 showed consistent activations in posterior parietal areas (SPL, IPL), and IPS. Finally, lobule VI of the cerebellum (bilaterally) was found consistently activated.

Multiple regression analysis between fMRI BOLD activation and MIA scores (temporal congruence between grasping action observation and imagination), obtained by UCP and TD children using the non-preferred hand, showed a significant effect in different areas associated to explicit MI (Figures 4C,D). Statistical details and MNI coordinates for local maxima of the regression effects in both groups are reported in Table 3.

**Table 3:** MNI coordinates and statistical details for brain areas showing a positive correlation with the MIA score obtained by UCP children in the mental chronometry task.

**Anatomical region**		**MNI coordinates**				
	**Side**	**x**	**y**	**z**	**Z-score**	**Cluster size**
**UCP CHILDREN**						
Intraparietal sulcus	LH*	−36	−44	+48	3.44	406
	RH	+32	−54	+52	4.00	377
Precentral gyrus	RH	+26	−4	+50	3.37	136
Putamen	RH	+26	−4	+6	4.16	145
Pallidum	RH	+18	−2	−2	4.14	
**TD CHILDREN**						
Superior parietal lobule	LH	−26	−62	+64	6.05	498
	RH	+22	−60	+56	5.57	374
Intraparietal sulcus	LH	−42	−42	+46	5.36	436
	RH	+30	−44	+44	5.62	348
Precentral gyrus	RH	+28	−2	+50	4.58	168
Supramarginal gyrus	RH	+60	−36	+30	4.06	131
IFG (pars opercularis)	RH	+54	+12	−2	4.34	198
Cerebellum (VI)	LH	−32	−60	−20	4.20	128
	RH	+30	−58	−20	4.22	102

*Significant threshold set at p < 0.001, FWE corrected at cluster level. Cluster size indicates voxel number. LH, Left Hemisphere; RH, Right Hemisphere. Asterisk indicates the lesioned hemisphere in UCP children*.

In the UCP group, the BOLD signal within ipsilesional and contralesional IPS was positively correlated with MIA scores obtained in mental chronometry task performed with the non-preferred hand (right IPS, x = +28, y = −56, z = +51; *r* = 0.92, *p* < 0.001; left IPS, x = −28, y = −60, z = +42; *r* = 0.93, *p* < 0.001). A similar result was present also in the contralesional PMd (right PMd, x = +34, y = +2, z = +52; *r* = 0.83, *p* < 0.01) and putamen (right putamen, x = +28, y = −12, z = +11; *r* = 0.61, *p* < 0.05). In the left ipsilesional hemisphere there were no further significant correlations with the MIA score related to the non-preferred hand.

Similarly, the regression analysis on TD group's activations revealed positive correlation between behavioral score and BOLD activity in several areas including, in the left hemisphere, the IPS bilaterally (left: x = −42, y = −42, z = +46; *r* = 0.87, *p* < 0.001; right: x = +30, y = −44, z = +44; *r* = 0.92, *p* < 0.001) and the SPL bilaterally (left: x = −26, y = −62, z = +64; *r* = 0.74, *p* < 0.001; right: x = +22, y = −60, z = +62; *r* = 0.77, *p* < 0.001).

In the right hemisphere, areas showing a positive correlation with MIA scores included the PMd cortex (x = +28, y = −2, z = +50; *r* = 0.81, *p* < 0.001), and IFG *pars opercularis* (x = +54, y = +12, z = −2; *r* = 0.65, *p* < 0.001). Interestingly, a positive correlation in TD group was found in the cerebellum (Lobule VI), bilaterally (left: x = +32, y = −60, z = −20; *r* = 0.83, *p* < 0.001; right: x = +30, y = −58, z = −20; *r* = 0.89, *p* < 0.001).

## Discussion

Here, we examined the explicit MI ability in children affected by spastic UCP and children with typical development. Participants were explicitly instructed to execute actions, consisting in grasping an object with their preferred or non-preferred hand and placing it into a box, or to imagine themselves perform the same action, from a first-person perspective. Furthermore, a subgroup of UCP children and a subgroup of TD children performed an explicit MI task during a session of fMRI, aimed at investigating functional activation associated to the imagination of grasping actions. Three main results became evident. First, UCP children demonstrated significant temporal correlation between durations of actual and imagined movements. It should be noted, however, that the durations of both action execution and imagination were significantly slower in UCP children compared to TD participants, both when patients performed the task with the preferred and the non-preferred hand. Secondly, the performance of UCP children was similar to that of age-matched TD children in terms of temporal congruence between duration of execution and imagination trials. However, not all UCP children presented a good correlation between execution and imagination. Thirdly, both the subgroups of UCP and TD children who underwent the fMRI experiment showed a positive correlation between brain activations during imagination of grasping movements and behavioral score obtained during the mental chronometry task using the non-preferred hand. In fact, higher scores in the behavioral test were positively correlated, in TD participants, to increased fMRI activations within the IPS, SPL, and Cerebellum, bilaterally, and the right PMd cortex. Similarly, in children with UCP, a positive correlation was found between behavioral score and BOLD activity in IPS bilaterally, and in contralesional right PMd cortex and putamen. These results will be discussed in more detail in the next sections.

### Explicit Motor Imagery for Grasping Actions in Children With UCP

In the past decade, several studies using implicit MI tasks like the HLJ, reported that MI is severely impaired in UCP children (12, 25, 26, 55, 56). On the other hand, recent works (31, 32) support the idea that explicit imagery processes increase body awareness and facilitate patients to use an effective motor strategy. Previous works in healthy individuals used mental chronometry tasks to study the development of MI (30, 33, 40) reporting an age-related increase in ability to engage an effective motor strategy during action imagination between 5 and 12 years (i.e., MI duration complies strongly with Fitts' law assumptions). In agreement with these studies in TD children, the present study demonstrates that also UCP children are capable of explicit MI, suggesting that explicit paradigms of imagination for grasping actions may reveal a MI capacity in these patients. To the best of our knowledge, only few previous studies employed an explicit paradigm to explore MI in children affected by spastic UCP (32, 57). For example, Spruijt et al. (32) investigated MI capacity in CP children by means of a mental chronometry task for walking, demonstrating that task difficulty has similar effects on movement durations for both actual walking and imagined walking. Here, we required children to imagine an action consisting in grasping an object and placing it into boxes, more complex than other classical tasks, like pointing movements, walking etc. Similarly to previous studies (34, 38, 58), we also manipulated task difficulty (41) introducing three different target distances in both real action execution and in imagination tasks.

Our findings indicate clearly that UCP patients, similarly to TD children, showed a preserved ability to imagine grasping actions, both with the preferred and the non-preferred hand, using a motor strategy, as shown by the compliance of movement duration with the increase in target distance. Note, however, that in both hand conditions, the duration of action execution and motor imagery was higher in UCP than in healthy participants. A possible interpretation for this result could be that UCP children generally show a deficit in the forward modeling of movements (12); this could make MI particularly difficult when instructions require an *implicit* simulation of the action. However, in our study, UCP children are likely facilitated by the *explicit* instruction to use MI of a goal directed sequence, which requires them to perform imagination in a first-person perspective, allowing them to evoke the same kinesthetic experience that characterize the real action execution. Thus, explicit MI instructions could favor UCP children performance, although this latter does not reach the level of TD children.

A further, not alternative, explanation for the longer duration of execution and MI, both for the non-preferred and the preferred hand, could be referred to a higher order deficit related to general action planning. Since the task required a three-step sequence, it is possible that the memory retrieval of the sequence and/or transition between its various steps require more time with respect to healthy controls. This explanation may indicate a possible bilateral deficit in action organization in UCP children (26, 59).

### Brain Activations During Explicit Motor Imagery in UCP and TD Children

According to behavioral findings obtained by the mental chronometry task, we found that some UCP children showed preserved brain activations in cortical and subcortical areas already described in previous studies on healthy individuals during performance of kinesthetic MI tasks (15). Activation of these areas was very similar to that of TD children performing the same MI task. It could be due to the employed modality of MI requiring the imagination of that movement in a first-person perspective, perceiving the motor action (e.g., retrieving sensations typically associated with object texture, proprioception etc.), that is different from other imagination strategies, such as visual imagery, consisting in mentally visualizing a movement in third-person perspective. Among the main activated regions in UCP children and healthy controls, premotor and parietal areas have been reported in previous studies, in which the task required healthy participants to execute (60–63), to observe (64–67), or to imagine (68–71) reaching-grasping actions.

The results of our study suggest that UCP children were able to activate, in the right contralesional hemisphere, a similar parieto-frontal network during imagination of reaching-grasping actions performed with the non-preferred hand. Interestingly, some UCP children (*N* = 4) showed enhanced brain activations not only in the contralesional hemisphere, but also in the ipsilesional one, including, in the parietal cortex, the IPS/SPL and SMG/IPL and, in the frontal cortex, the PMd. Noteworthy, these are the patients in which the damage was mainly subcortical. Moreover, two of these patients (#2, #6) showed activations also in the ipsilesional prefrontal region (DLPFC).

These findings are partly in contrast with other studies on MI in UCP children (25, 26, 72), which suggest a general deficit in MI and motor planning for hemiparetic CP children with motor deficits on the right side compared with the left one. For instance, Chinier et al. (72) showed that only UCP patients with early lesions of the right hemisphere were able to perform MI. A plausible explanation for this discrepancy could be the specificity of MI modality, explicitly requiring participants to imagine themselves performing the action in a first-person perspective.

One can argue that residual MI ability in UCP children of our study could be attributed to differences in lesion extension. By using lesion overlap, we found that UCP children enrolled in this study presented unilateral brain lesions involving the periventricular zone, with the greatest overlap in subcortical white matter of left hemisphere. Conversely, cortical involvement of regions outside the periventricular zone, i.e., inferior frontal, dorsolateral frontal, inferior and superior parietal regions, was much less frequently found (*N* = 2 patients). However, we did not find a direct correspondence between lesion type/extension and brain activation.

Another interesting result was that in both UCP and TD children the MIA scores obtained in the mental chronometry task using the non-preferred hand were correlated with BOLD activation during the covert execution of reaching-grasping actions with the same hand. In this respect, the activation of bilateral IPS and SPL in TD children during the imagination of grasping actions is in line with previous results on adults suggesting an important role of the parietal cortex during explicit MI (15, 69). Furthermore, deficits in MI tasks have been observed in patients with lesions of the parietal region (38).The correlation of behavioral score with IPS activation in UCP during MI performed with the paretic hand would support the idea that also in patients the imagination of reaching-grasping motor acts automatically retrieves the internal motor representation (13). Another possible interpretation could be that, since the task required children to imagine performing a complex action with their non-preferred hand, IPS activation may also be due to the online control necessary for correct execution.

The correlation of behavioral scores with activation of dorsal sector of premotor cortex (PMd), both in TD and UCP children, could be related to its role in online control of action execution. For example, a study in healthy participants, performing a reaching-grasping-lifting task, showed that TMS virtual lesions of PMd delayed recruitment of proximal muscles involved in the lifting phase, leading to a longer preloading phase and a less synchronized grasping/lifting movement (73), therefore suggesting an important role of PMd in synchronization of proximal and distal movements. The hand type of UCP children enrolled in this study requires, for grasping accomplishment, a synergy of shoulder, elbow and hand movements, exclusively under visual control. The same type of synergy could occur during imagination using the non-preferred hand. Consistent with this idea, activation of PMd cortex was stronger in those UCP children who obtained higher scores in the mental chronometry task.

Finally, the present data support also the view that subcortical regions, such as putamen and cerebellum, are involved in explicit MI for complex grasping actions. All cortical areas activated during imagination (IPL, IPS, PMd) are known to have strong anatomical connections with basal ganglia, creating a cortico-basal ganglia-thalamo-cortical loop that plays a key role in motor planning and motor learning (74). Furthermore, neuroimaging studies show activation of basal ganglia in healthy participants during MI (15). Coherent with this, patients with Parkinson's disease, which affects primarily the basal ganglia, show a deficit in mental representations of hand movements (75, 76). Based on this assumption, it is possible to hypothesize that in UCP children the normal motor loop connecting cortical motor system with basal ganglia via thalamus is involved in the performance of both overt and covert movement execution.

## Conclusions

This study substantiates the view that explicit MI ability for grasping actions could be preserved in UCP children, as demonstrated by temporal correlation between durations of actual and imagined movements. Brain activations related to explicit MI support this view. In summary, specific tools for MI assessment are necessary in order to evaluate the spared ability, which allows UCP children to retrieve motor representations, according to their residual motor repertoire. In addition, our data suggest that employing explicit MI strategies as a training tool could support, in CP rehabilitation, recovery of manipulation function and development of upper limb skilled movements. This suggestion is also prompted by the well-known evidence that MI can be used for rehabilitation of motor deficits, for example in developmental coordination disorder, in stroke patients and in Parkinson's disease (19–23). In addition, there is evidence that the use of MI in combination with Action Observation can represent an effective tool to enhance motor learning and improve upper limb function in patients with motor deficits (77–80). The employment of MI together with electrophysiological methods and behavioral scales who provide a better characterization of the patient, could allow to choose the type of MI (explicit vs. implicit) and of MI task (simple vs. complex actions) more appropriate for personalized interventions.

## Data Availability

All datasets analyzed for this study are included in the manuscript and Supplementary Files.

## Ethics Statement

Written informed consent was obtained from children, parents prior to recruitment, according to the Declaration of Helsinki. This study has been approved by the Institutional Review Board (IRB) of the University of Parma and by the local ethics committee (Hospital and University of Parma).

## Author Contributions

AE, AF, and LF contributed to experimental design. AE, SS, and BB performed the behavioral experiment. FB and GC performed the neuroradiological investigation. AE performed fMRI data acquisition, analyzed the behavioral and fMRI data. AE, FB, SS, SC, GC, AF, and LF wrote the manuscript.

### Conflict of Interest Statement

The authors declare that the research was conducted in the absence of any commercial or financial relationships that could be construed as a potential conflict of interest.

## Abbreviations

AON: Action Observation Network
CP: Cerebral Palsy
HFC: House Functional Classification
MACS: Manual Ability Classification System
MI: Motor Imagery
MNS: Mirror Neuron System
UCP: Unilateral Cerebral palsy.
