# Supplementary material for: Explicit Motor Imagery for Grasping Actions in Children With Spastic Unilateral Cerebral Palsy
Source: Front Neurol. 2019 Aug 7;10:837. doi: 10.3389/fneur.2019.00837 (PMC6692593; doi:10.3389/fneur.2019.00837)
Supplement: Supplementary file 1 [file Table_1.DOCX]

| UCP | EXE Dis:100  Preferred Hand | EXE Dis:150  Preferred Hand | EXE Dis:200  Preferred Hand | EXE Dis:100  Non-Preferred Hand | EXE Dis:150  Non-Preferred Hand | EXE Dis:200  Non-Preferred Hand |
| --- | --- | --- | --- | --- | --- | --- |
| #1 | 2501 | 2989 | 3554 | 3267 | 3366 | 4179 |
| #2 | 2186 | 3890 | 2364 | 2847 | 5527 | 3512 |
| #3 | 2049 | 2079 | 2666 | 2754 | 2873 | 3054 |
| #4 | 1431 | 3182 | 3247 | 1982 | 3209 | 4410 |
| #5 | 1873 | 2572 | 3998 | 2813 | 3310 | 4300 |
| #6 | 1899 | 1996 | 2340 | 2284 | 2735 | 2904 |
| #7 | 2253 | 2912 | 3469 | 4066 | 4998 | 5876 |
| #8 | 1163 | 1136 | 1406 | 1901 | 2076 | 2254 |
| #9 | 1760 | 2224 | 3013 | 2884 | 4605 | 5786 |
| #10 | 2221 | 3437 | 3264 | 2979 | 3966 | 4123 |

| UCP | MI Dis:100  Preferred Hand | MI Dis:150  Preferred Hand | MI Dis:200  Preferred Hand | MI Dis:100  Non-Preferred Hand | MI Dis:150  Non-Preferred Hand | MI Dis:200  Non-Preferred Hand |
| --- | --- | --- | --- | --- | --- | --- |
| #1 | 2674 | 2913 | 3331 | 2940 | 3766 | 4216 |
| #2 | 2663 | 4519 | 2404 | 3396 | 2446 | 3013 |
| #3 | 2283 | 2625 | 2631 | 4765 | 3226 | 3318 |
| #4 | 1319 | 2093 | 3607 | 2548 | 2748 | 3124 |
| #5 | 1981 | 2655 | 2975 | 2292 | 2855 | 2479 |
| #6 | 1823 | 1727 | 2321 | 3159 | 2240 | 2901 |
| #7 | 3061 | 5215 | 6068 | 3641 | 5196 | 3204 |
| #8 | 1923 | 2067 | 1977 | 2371 | 2746 | 2834 |
| #9 | 3046 | 3247 | 3767 | 4045 | 4127 | 4843 |
| #10 | 2680 | 3516 | 3749 | 3403 | 3801 | 4399 |

Suppl. Table 1: Mean duration for each UCP patient during the performance of Action Execution (EXE) and Motor Imagery (MI) tasks.
