# Supplementary material for: Explicit Motor Imagery for Grasping Actions in Children With Spastic Unilateral Cerebral Palsy
Source: Front Neurol. 2019 Aug 7;10:837. doi: 10.3389/fneur.2019.00837 (PMC6692593; doi:10.3389/fneur.2019.00837)
Supplement: Supplementary file 2 [file Table_2.DOCX]

| Sub. # | Sex | Age | Group | CP Type | HFC | MACS | MIA Preferred hand | MIA Non-Preferred Hand |
| --- | --- | --- | --- | --- | --- | --- | --- | --- |
| 1 | M | 10 | TD | - | - | - | 0,48 | 0,48 |
| 2 | F | 10 | TD | - | - | - | 0,66 | 0,43 |
| 3 | F | 11 | TD | - | - | - | 0,72 | 0,64 |
| 4 | M | 11 | TD | - | - | - | 0,67 | 0,84 |
| 5 | F | 11 | TD | - | - | - | 0,74 | 0,13 |
| 6 | F | 11 | TD | - | - | - | 0,72 | 0,29 |
| 7 | F | 11 | TD | - | - | - | 0,5 | 0,69 |
| 8 | F | 12 | TD | - | - | - | 0,69 | 0,51 |
| 9 | F | 12 | TD | - | - | - | 0,57 | 0,45 |
| 10 | M | 14 | TD | - | - | - | 0,66 | 0,12 |
| 11 | F | 14 | TD | - | - | - | 0,46 | 0,23 |
| 12 | M | 11 | TD | - | - | - | 0,68 | 0,62 |
| 1 | F | 13 | UCP | Right UCP | 5 | 3 | 0,64 | 0,55 |
| 2 | M | 14 | UCP | Right UCP | 5 | 3 | 0,52 | 0,31 |
| 3 | M | 9 | UCP | Right UCP | 5 | 3 | 0,55 | 0,33 |
| 4 | F | 13 | UCP | Right UCP | 4 | 3 | 0,62 | 0,54 |
| 5 | M | 10 | UCP | Right UCP | 5 | 3 | 0,42 | 0,25 |
| 6 | M | 10 | UCP | Right UCP | 5 | 3 | 0,57 | 0,39 |
| 7 | M | 14 | UCP | Right UCP | 5 | 3 | 0,59 | 0,44 |
| 8 | F | 13 | UCP | Left UCP | 5 | 2 | 0,24 | 0,15 |
| 9 | F | 14 | UCP | Left UCP | 4 | 3 | 0,32 | 0,26 |
| 10 | F | 13 | UCP | Left UCP | 5 | 2 | 0,65 | 0,16 |

Suppl. Table 2. Individual Motor Imagery Ability scores obtained by each TD and UCP participant using the preferred and the non-preferred hand.
