# Supplementary material for: Explicit Motor Imagery for Grasping Actions in Children With Spastic Unilateral Cerebral Palsy
Source: Front Neurol. 2019 Aug 7;10:837. doi: 10.3389/fneur.2019.00837 (PMC6692593; doi:10.3389/fneur.2019.00837)
Supplement: Supplementary file 3 [file Image_1.pdf]

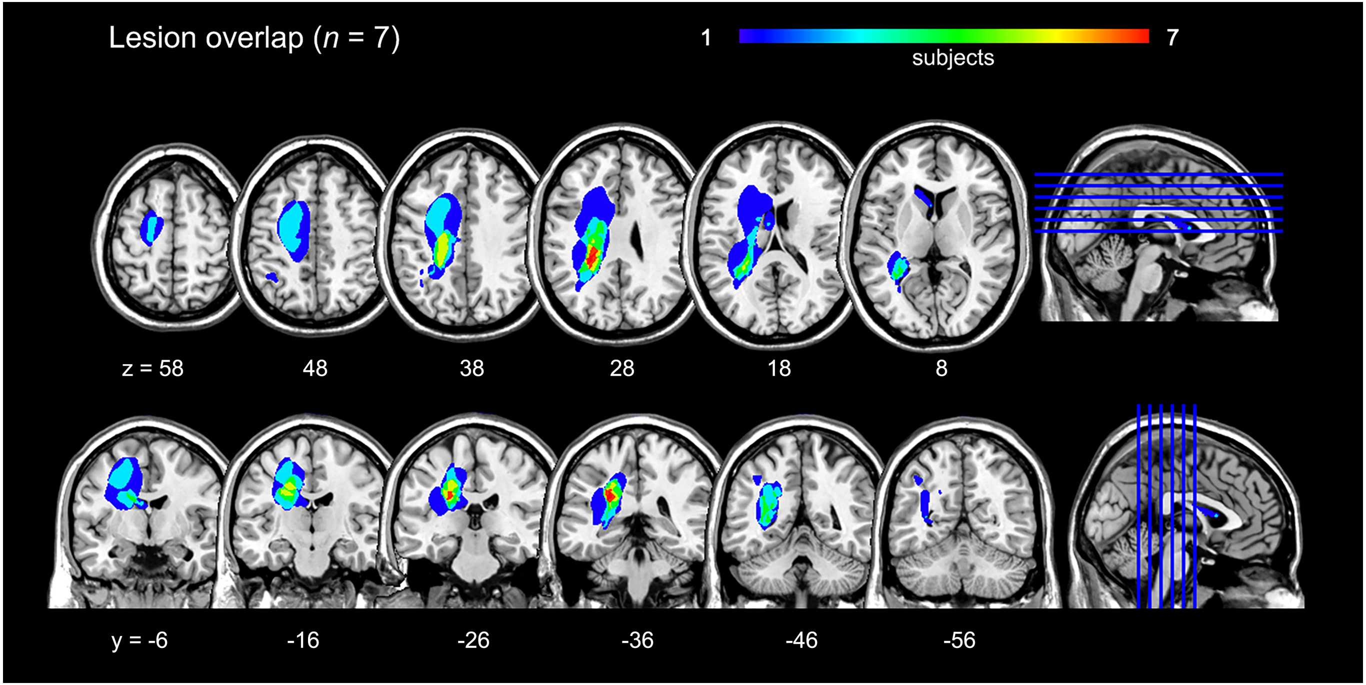

Suppl. Fig. 1. Overlap of the binarized lesion maps of the 7 UCP patients involved in the MRI study. The colour bar indicates the degree of overlap of lesions, e.g. red colour indicates the maximum overlap. Lesions are overlaid onto the surface of a standard MNI template (ch2Better, MRICron).
